# Asbestos Consumption and Malignant Mesothelioma Mortality Trends in the Major User Countries

**DOI:** 10.5334/aogh.4012

**Published:** 2023-02-13

**Authors:** Claudio Gariazzo, Antonio Gasparrini, Alessandro Marinaccio

**Affiliations:** 1 Occupational and Environmental Medicine, Epidemiology and Hygiene Department, Italian Workers’ Compensation Authority (INAIL), Roma, Italy; 2 Department of Public Health Environment and Society, London School of Hygiene & Tropical Medicine, London, United Kingdom

**Keywords:** mesothelioma, asbestos, mortality, international ban, distributed non-linear models

## Abstract

**Background:**

The causal association between mesothelioma and asbestos exposure is conclusive, and many studies have proved that the trend in asbestos use is a strong predictor of the pattern in mesothelioma cases with an adequate latency time (generally around 30–40 years or more). Recently, a novel approach for predicting malignant pleural mesothelioma, based on asbestos consumption trend and using distributed non-linear models, has been applied.

**Objectives:**

The purpose of this study is to analyse trends in asbestos consumption and malignant mesothelioma mortality in the major asbestos-user countries. Furthermore, we applied distributed non-linear models to estimate and compare epidemiological relationships between asbestos consumption and mesothelioma mortality across these countries.

**Methods:**

The study involves major asbestos-user countries in which historical asbestos consumption and mesothelioma mortality data are available. Data on asbestos consumption were derived from worldwide asbestos supply and mesothelioma mortality data from World Health Organization (WHO) mortality archives. A quasi-Poisson generalized linear model was used to model past asbestos exposure and male mesothelioma mortality rates in each country. Exposure-response associations have been modelled using distributed lag non-linear models.

**Findings and conclusions:**

According to the criteria defined above, we selected 18 countries with raw asbestos cumulative consumptions higher than two million tons in the period 1933–2012. Overall, a clear linear relationship can be observed between total consumption and total deaths for mesothelioma. Country-specific exposure, lag and age-response relationships were identified and common functions extracted by a meta-analysis procedure. Non-linear models appear suitable and flexible tools for investigating the association between mesothelioma mortality and asbestos consumption. There is a need to improve the global epidemiological surveillance of asbestos-related diseases, particularly mesothelioma mortality, and the absence of reliable data for some major asbestos-user countries is a real concern. A reliable assessment of mesothelioma mortality is a fundamental step towards increasing the awareness of related risks and the need of an international ban on asbestos.

## Introduction

According to the Global Burden of Diseases, Injuries, and Risk Factors Study 2017, exposure to asbestos fibres causes more than 250,000 deaths annually [1]. The International Agency for Research on Cancer (IARC) confirmed that all forms of asbestos are carcinogenic for humans (group 1) causing mesothelioma, as well as lung, larynx, and ovary cancer. Furthermore, positive associations have been observed between exposure to all forms of asbestos and pharynx, stomach, and colorectal cancers with limited evidence [2]. The causal association between mesothelioma and asbestos exposure is conclusive, and many studies have proved that the trend in asbestos use is a strong predictor of the pattern in mesothelioma cases with an adequate latency time (generally around 30–40 years or more) [3]. There is solid evidence that the best way to avoid asbestos-related diseases is to ban asbestos, and the World Health Organization (WHO), the International Labour Organization (ILO) [4], and Collegium Ramazzini [5] have strongly advocated for ceasing the use of asbestos. Nevertheless, asbestos consumption remains approximately constant at around two million metric tons per year worldwide at the present [6].

Recently, a novel approach for predicting malignant pleural mesothelioma mortality, based on asbestos consumption trend and using distributed non-linear models, has been applied using data for Italy [7], which was a great producer and asbestos consumer country until the ban in 1992 [8].

The estimation of asbestos-related diseases is essential for monitoring the consequences of asbestos use in countries where it has been banned, and in countries where asbestos is still used for industrial activities and present in occupational settings, as well as the environment. According to the US Geological Surveys [6], the leading consumers of asbestos in 2003 were Brazil, China, India, Iran, Kazakhstan, Russia, Thailand, and Ukraine, each consuming more than 75,000 tons/year [6]. Mesothelioma mortality data are not completely available for many countries and the health impact of asbestos use remains partially unknown.

The purpose of this study is to analyse trends in asbestos consumption and malignant mesothelioma mortality in the major asbestos-user countries. Furthermore, we applied distributed non-linear models to estimate and compare epidemiological relationships between asbestos consumption and mesothelioma deaths across these countries.

## Methods

### Study setting

The study involves major asbestos-user countries in which historical asbestos consumption and mesothelioma mortality data are available. Males are a target population of this study, as the main gender involved in occupational exposure and related health effects. Being based on country specific consumption data, the study addressed asbestos exposure as a whole, without distinguishing it between occupational and environmental sources, using a longitudinal approach by calculating exposure history with a pre-defined lag period with a further limitation on the working age population. Next, we modelled the relationship between past asbestos consumption and mesothelioma mortality to estimate country-specific exposure-response, lag-response and age-response functions.

### Asbestos consumption data

Data on asbestos consumption were derived from worldwide asbestos supply and consumption estimations provided by the United States Geological Surveys (USGS) technical reports [6], which include historical country-specific data of national production, import, and export based on information extracted from a variety of sources. Asbestos apparent consumption has been defined as domestic production plus the difference between imports and exports.

As a first step, we selected the major user countries of raw asbestos according to international trade figures, considering all countries with more than two million of raw asbestos consumption in the period 1933–2012. For descriptive trend analysis, we have divided this period into four 20-year sub-periods (1933–1952; 1953–1972; 1973–1992; 1993–2012), analysing the temporal distribution of asbestos consumption, comparatively by country.

### Mesothelioma mortality data

Malignant mesothelioma mortality has been retrieved from the WHO mortality database [9]. For each selected country, we have considered the number of males who died from mesothelioma (ICD-10: C.45) in a 20-year period (1998–2017). Russia, China, and India reported no mesothelioma death in the WHO mortality database archives, while data from Brazil were only partially available (not national data). Thailand presented 31 male deaths for mesothelioma in the entire period. For all other selected countries, we calculated the percentage variation in the four, five-year periods. As for the modelling of the association between consumption and mesothelioma mortality, we used yearly data from 1920 to 2018. For modelling the exposure, lag, and age-response functions, mortality data available in five-year age classes were interpolated obtaining occurrences for each yearly age.

### Male population data

Male population data during the period of 1950–2017 were retrieved from Global World Statistics 2022, which is based on data provided by World Bank and United Nation Organization [10]. Data are provided in five age classes (0–19; 20–39; 40–59; 60–79; 80+) and were interpolated at an yearly resolution in the interval 0–100 years of age. The total population, needed to calculate per capita asbestos consumption, was interpolated up to the year 1920 using historical data.

### Asbestos exposure

To estimate the lifetime exposure to asbestos of the population, we assigned the asbestos consumption per capita by year during the period of 1920–2018 to the whole population regardless of age. We calculated the related exposure history with a pre-defined lag period, setting lag 0 equal to the exposure that occurred during the last year. Therefore, assuming a maximum lag of 60 years, we generated a matrix of exposure histories of the population for each age and year that was used to define temporal dependencies using the model presented in the next section. We assumed that asbestos consumption before 1920 was equal to that estimated for this year. Finally, we assumed that occupational exposure to asbestos was limited to the working age (15–65 years old). The matrix of historical asbestos exposure was then merged by age and year with mesothelioma mortality to fit the statistical model. Modelling was restricted to the countries with reliable mortality data (12 of the 18 original countries). China, Russia and India were discarded due to the lack of mesothelioma mortality data in the WHO database. Thailand and Brazil were discarded due to incompleteness in mortality data, and Mexico for lack of consistency in the asbestos exposure range with respect to other countries. The model was applied to male subjects of age 25–89, considered as those likely involved with mesothelioma mortality due to past asbestos exposure.

### Statistical model

We extended the modelling approach used in a study carried out in Italy [7] to determine the association between past asbestos consumption and mesothelioma male mortality, applying it in a multi-country context using a two stages analysis. We have modelled only mesothelioma deaths in men in non-linear models, according to the less straightness of the association in females. In the first stage, we fitted a quasi-Poisson generalized linear model with age/year-specific mesothelioma deaths to model the association between past asbestos exposure and mesothelioma mortality rates in each country.

The model is defined in the equation below:


1 \log \left[ {E\left( {{Y_t}} \right)} \right] = \alpha + f\left( {{E_t};\,\theta } \right) + g\left( {ag{e_t};\beta } \right) + \log \left( {po{p_t}} \right)

Where the outcome *Y_t_* corresponds to yearly counts of mesothelioma deaths assumed to follow a Poisson distribution with over dispersion, the function *f*(*E_t_*;θ) specifies the association with the asbestos exposure at year *t*, the function g\left( {ag{e_t};\beta } \right) specifies the association with age of the subjects at year *t*, and *log(pop)* is the corresponding log-transformed population at year t used as an offset.

The exposure–response associations have been modelled using distributed lag non-linear models (DLNM), a statistical framework to represent complex exposure–lag–response risk surfaces [11]. The function f(E_t_;θ) is defined by a bi-dimensional cross-basis term, using a natural spline function with two degrees of freedom and equally spaced knots for the exposure–response dimension, and a natural spline function with three degrees of freedom to model the lagged-response dimension, accounting for 60 years of lag. The age-response function g\left( {ag{e_t};\beta } \right) was represented by a one-dimensional natural spline function with a knot placed at age 65. In the second stage, the cross-basis coefficients obtained for each country were then used to obtain an overall pooled exposure-response function using mixed-effects meta-analysis [12]. We also estimated the best linear unbiased estimators (BLUPs) to obtain country-specific exposure response functions. The meta-analysis was also carried out for both lag-response and age-response functions defined at specific exposure and lag values, respectively. A sensitivity analysis about the dependence of the meta-analysed exposure, lag, and age-response curves, using different degrees of freedom of the natural splines functions, has been carried out.

## Results

### Trend analysis of asbestos consumption and mesothelioma mortality

According to the criteria defined above, we selected 18 countries with raw asbestos cumulative consumptions higher than two million tons in the period 1933–2012. Eight countries in Europe (Belgium, France, Germany, Italy, Poland, Russia, Spain, and the United Kingdom), five in Asia (China, India, Japan, South Korea, and Thailand), four in North and South America (Brazil, Canada, Mexico, and the USA), together with Australia. According to USGS technical reports, these countries represented 79% of total asbestos consumption in the world during the study period [6].

Table 1 shows the asbestos consumption in each country during four, twenty-year periods and the percent of variation with respect to the previous one. The huge increase in asbestos consumption in the period 1953–1972 is noteworthy, with respect to the previous 20-year period in all countries. In the subsequent 20 years (1973–1992), some countries registered a reduction in asbestos consumption: Canada and United States of America (–11% and –58, respectively), Belgium, France, and the United Kingdom in Europe (–21%, –5% and –56%, respectively), and Australia (–23%). In 1993–2012, all countries presented a decrease in asbestos consumption from the previous period, with the exception of China, India, and Thailand, which consistently still increased the use of asbestos (161%, 127% and 64%, respectively).

**Table 1:** Asbestos consumption for selected countries (countries with more than two million tonnes of asbestos consumption in the period 1933–2012) in four 20-year periods, and percentage of variation by period and country.


REGION	COUNTRY	ASBESTOS CONSUMPTION (TONS)							

		OVERALL (1933–2012)	1933–1952	1953–1972		1973–1992		1993–2012	
		
					VARIATION [%]		VARIATION [%]		VARIATION [%]

America	Brazil (BZ)	6,702,392	80,684	609,435	655	3,006,729	393	3,005,545	0

America	Canada (CAN)	3,149,032	573,012	1,212,992	112	1,077,619	–11	285,408	–74

America	Mexico (MX)	2,088,301	53,393	460,796	763	1,143,907	148	430,205	–62

America	United States of America (US)	26,441,639	7,784,427	13,027,033	67	5,515,179	–58	115,000	–98

Asia	China (CN)	15,957,810	27,361	2,070,665	7,468	3,843,034	86	10,016,751	161

Asia	India (IN)	7,043,272	141,417	641,333	354	1,916,880	199	4,343,643	127

Asia	Japan (JP)	11,209,668	405,977	3,289,065	710	6,036,293	84	1,478,333	–76

Asia	Republic of Korea (ROK)	2,139,073	60,503	297,593	392	1,222,174	311	558,803	–54

Asia	Thailand (TH)	4,091,998	–	207,671	–	1,473,500	610	2,410,827	64

Europe	Belgium (BE)	1,956,961	213,121	949,752	346	746,671	–21	47,417	–94

Europe	France (FR)	4,672,674	518,213	2,051,029	296	1,943,113	–5	160,319	–92

Europe	Germany (DE)	9,202,903	872,733	3,677,755	321	4,640,044	26	12,371	–100

Europe	Italy (IT)	4,513,664	350,757	1,773,280	406	2,389,407	35	220	–100

Europe	Poland (PL)	2,460,804	54,276	663,007	1,122	1,585,806	139	157,714	–90

Europe	Russia (RU)	45,344,666	1,494,255	7,989,488	435	26,721,566	234	9,139,357	–66

Europe	Spain (ES)	2,190,005	72,072	739,022	925	1,173,815	59	205,096	–83

Europe	United Kingdom (UK)	6,177,405	1,810,254	2,999,349	66	1,318,343	–56	49,459	–96

Oceania	Australia (AU)	2,061,747	288,057	995,438	246	765,180	–23	13,072	–98


Table 2 shows the number of persons who died from malignant mesothelioma in major asbestos-user countries during four periods of five years between 1998 and 2017. Data about China, India, and Russia are missing, as data were not available in the WHO mortality database archives. Thailand is not present in Table 2 due to the inconsistency of reported cases (31 male deaths due to mesothelioma in the period). Most of the selected countries presented an increase in the number of cases by five-year period, with a decreasing trend in percentage variation (Belgium, Canada, France, Germany, Italy, Japan, Mexico, and South Korea). The USA is the only country for which a decrease in the number of deceased subjects is documented in the last period with respect to the previous one (13,056 deaths for mesothelioma in 2008–2012, and 12,659 in 2013–2017). These findings support the positive effects of asbestos ban (or restrictions policies) on mesothelioma mortality trend. Brazil, which included data only for a region, presented an irregular trend with a first decrease between 1998–2002 and 2003–2007 (558 and 366 deaths, respectively) and a clear increasing trend subsequently (412 cases in 2008–2012 and 508 in the subsequent period).

**Table 2:** Number of mesothelioma deaths for selected countries (with more than 2 million tonnes of asbestos consumption in the period 1920–2012) by period and percentage of variation.


FIVE-YEAR PERIOD	AUSTRALIA	BELGIUM	BRAZIL	CANADA	FRANCE	GERMANY	ITALY	JAPAN	MEXICO	POLAND	REPUBLIC OF KOREA	SPAIN	UNITED KINGDOM	UNITED STATES OF AMERICA

1998–2002	2,237	821	558	1,482	3,792	5,037	5,095	3,509	620	308	113	1,251	8,434	11,923

2003–2007	2,621	961	366	1,852	4,292	5,757	6,025	4,860	893	513	232	1,440	9,665	12,417

Variation [%]	17	17	–34	25	13	14	18	39	44	67	105	15	15	4

2008–2012	3,093	1,104	412	2,176	4,920	6,729	6,968	6,193	1,134	924	384	1,904	11,481	13,056

Variation [%]	18	15	13	17	15	17	16	27	27	80	66	32	19	5

2013–2017	3,393	1,225	508	2,489	5,025	7,186	7,950	7,395	1,332	1,512	508	2,148	12,715	12,659

Variation [%]	10	11	23	14	2	7	14	19	17	64	32	13	11	–3

Total	11,344	4,111	1,844	7,999	18,029	24,709	26,038	21,957	3,979	3,257	1,237	6,743	42,294	50,055


**Figure 1:**
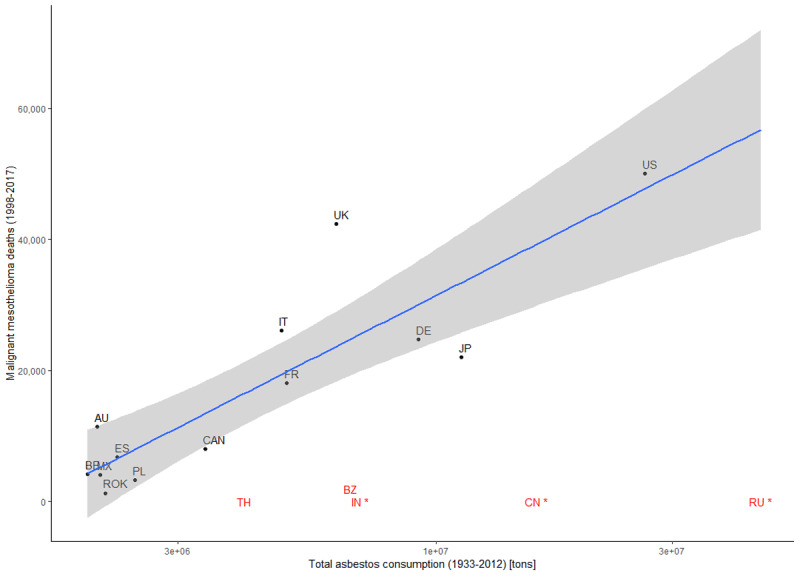
Overall asbestos consumption (1933–2012) and malignant mesothelioma deaths (1998–2017) for selected countries. Regression curve and 95% confidence interval. In red, countries with incomplete mortality data, with asterisk countries missing mortality data. Country abbreviations are the following: Australia (AU), Belgium (BE), Brazil (BZ), Canada (CAN), China (CN), Germany (DE), Spain (ES), France (FR), India (IN), Italy (IT), Japan (JP), Mexico (MX), Poland (PL), Republic of Korea (ROK), Russia (RU), Thailand (TH), United Kingdom (UK), United States of America (US).

Figure 1 tabulates the association between asbestos consumption (1933–2012) and mesothelioma deaths (1998–2017), showing the distribution of countries. Russia, China, and India have been tabulated with no mesothelioma cases registered. A clear linear relationship can be observed between total consumption and total deaths for mesothelioma.

### Results of the statistical models

**Figure 2:**
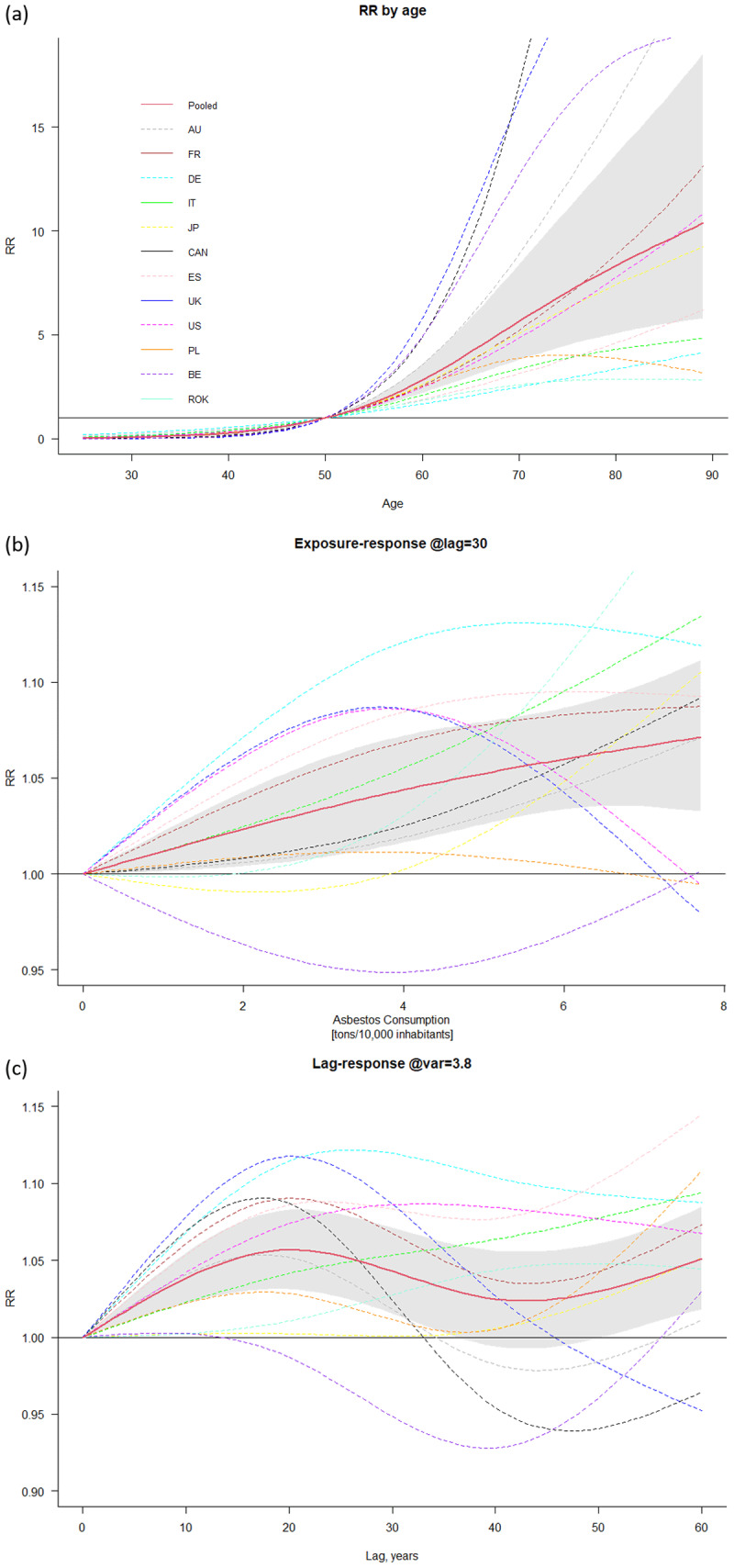
Country-specific relative risk **(RR)** of malignant mesothelioma and pooled meta-analytical curves (in red) **and 95% confidence intervals** (in grey) by age (upper), asbestos consumption at lag 30 years (middle), lag at 3.8 tons/10,000 inhabitants of asbestos exposure (bottom). **Baselines for RR are set to 50 years for age, and 0 for both exposure-response and lag-response**. Country abbreviations are the following: Australia (AU), Belgium (BE), **Canada (CAN)**, Germany (DE), Spain (ES), France (FR), Italy (IT), Japan (JP), Poland (PL), **Republic of Korea (ROK)**, United Kingdom (UK), United States of America (US).

Country-specific and pooled meta-analytical exposure, lag, and age-response relationships are shown in Figure 2. High heterogeneity is observed among the countries. The overall pooled exposure-response function (Figure 2b) exhibits an increasing risk for asbestos consumption level. Heterogeneity is also detected among the country-specific lag-response functions (Figure 2c). The overall pooled lag-exposure response (latency) for an exposure of 3.9 tons for 10,000 inhabitants shows an interesting pattern. The lag-specific RR grew almost linearly for 20 years since exposure, followed by a drop which reflects the behaviour of some countries. The age-response relationship (Figure 2a) shows a slow increase until age 50 and a sharp increase thereafter with a large scatter for each countries. Figure SM1 displays the full exposure-lag-response through the bi-dimensional representation of the predicted risk over a grid of exposures and lags, obtained from pooled meta-analysis of country-specific results. Yearly country-specific model estimated mesothelioma deaths in men and cases registered in the WHO archive were compared to obtain a goodness of fit of the country model. Cumulative observed and predicted cases, mean squared error (MSE), and Spearman correlation coefficient of yearly data are presented in Table 3 for the years of data availability. The country-specific models show a good ability to reproduce the total observed malignant mesothelioma cases with a small bias. Low MSE values and a good correlation with observed values are also identified.

**Table 3:** Country specific model performances: male population (average in the observed period); observed and predicted malignant mesothelioma cases for males in the period of data availability (Years), Mean Squared Error (MSE), Spearman correlation coefficients between observed and predicted cases by year.


COUNTRY CODE			MALIGNANT MESOTHELIOMA CASES			
		
	MEAN MALE POPULATION (1950–2018)	YEARS	OBSERVED	PREDICTED	MEAN SQUARED ERROR (MSE)	SPEARMAN COEFFICIENT

AU	7,912,528	1998–2018	9,573	9,150	19.4	0.96

FR	26,379,622	2000–2014	10,240	9,923	26.7	0.81

DE	37,741,395	1998–2018	20,461	19,950	46.6	0.92

IT	27,017,583	2003–2017	15,035	14,642	30.0	0.96

JP	55,511,392	1995–2018	20,027	19,360	25.6	0.99

CAN	13,136,991	2000–2017	6,125	6,067	10.4	0.98

ES	18,270,966	1999–2017	4,892	4,441	16.6	0.94

UK	27,935,742	2001–2016	29,241	28,378	27.3	0.99

US	122,640,854	1999–2017	37,875	36,155	62.4	0.61

PL	16,916,318	1999–2018	2,385	2,358	10.0	0.98

BE	4,863,004	1998–2016	3,177	3,091	11.6	0.86

ROK	19,378,296	1995–2018	954	945	5.3	0.97


The exposure, lag, and age response curves obtained in the main analysis were tested for sensitivity to different values of the parameters used in the natural spline functions included in the cross-basis terms of the model. We tested different degrees of freedom for exposure and lag functions, and different number of knots for the age function. Results are shown in Supplemental Material (Figure SM2). In summary, the number of degree of freedom of the exposure function introduces minimal differences both in the exposure and the lag response curves. Similarly, minimal modifications are estimated when the number of degrees of freedom are increased in the lag function. Higher variations in the age-response curves are instead estimated as far as the number of knots in the natural spline function for age is increased. However, such differences are within the 95% confidence interval of the main analysis results.

## Discussion

The relation between the asbestos consumption trend and mesothelioma mortality after an adequate latency period (generally 30–40 years) has been analysed repeatedly in international comparative studies [3,13,14,15,16] and at national level [17,18,19,20] using traditional age-period-cohort models or other similar approaches. In this study, we have used longitudinal mesothelioma mortality retrieved from the WHO data for evaluating the association with historical asbestos consumption and comparing results across countries, using non-linear models. The advantages of using distributed lag non-linear models (DLNM) are the possibility to include in one model the demographic information (age, period, birth cohort) and, at the same time, to use asbestos consumption as a predictive variable, dealing with non-linarites relationships between exposure and health outcomes. Furthermore, non-linear models allow considering the whole period of latency (from the exposure beginning to the health outcome), rather than fixing latency time as in many traditional linear models. In general, non-linear models appear more suitable and flexible for investigating prediction scenarios in ecological studies when demographic and exposure variables have to be used simultaneously. In this study, we have used longitudinal mesothelioma mortality retrieved from the WHO data for evaluating the association with historical asbestos consumption and comparing results across countries. This allowed evaluating the legacy of asbestos use in a cost-benefits perspective and the completeness of available data about asbestos-related health effects. Our results emphasized the inconsistency of mesothelioma mortality data for many great asbestos-user countries not reported in the WHO mortality database, supporting the need to improve the epidemiological surveillance of asbestos-related diseases worldwide. Particularly, China, India and Russia do not register mesothelioma deaths, despite historical asbestos consumption trends induced to estimate approximatively 40,000, 20,000 and 50,000 expected mesothelioma deaths in males in the period 1998–2017, respectively. For some other countries, the number of deaths is partial (e.g., Brazil) or inconsistent with asbestos consumption (e.g., Thailand). The absence or inconsistency of any form of epidemiological surveillance of asbestos-related health effects could induce scarce awareness of the risk in the workers and communities, as well as barriers in detecting and removing potential sources of exposure.

The present study performed a country-specific analysis for modelling exposure-response by non-linear models for the most world asbestos consumers in a comparative context. Furthermore, from a public health point of view, we have provided evidence of the inconsistency of data about mesothelioma deaths in some of the greatest asbestos user countries, highlighting the substantial role of the epidemiological surveillance of asbestos-related diseases for correctly evaluating the health effects of asbestos use.

Despite the high heterogeneity in the country-specific exposure-lag-responses, this study estimated overall response functions using meta-analytical methods. Results suggested an almost linear increase of risk with asbestos consumption, after about 20 years and an indication of an increased risk even at higher latency years. The large confidence intervals indicate low accuracy, likely due to imprecision in both asbestos consumption and mesothelioma mortality data.

The major limitation of this study is the use of asbestos consumption as a proxy for asbestos exposure at national level. This assumption could be affected by bias according to a set of not available information. The relationship between consumption and exposure can be affected by the number of exposed workers, the level of environmental contamination, the type of asbestos (crocidolite or chrysotile, above all), the preventive measures adopted for protecting workers, and finally the modalities and the economic activities of asbestos use. These variables could have a substantial role in modelling the relationship between consumption and exposure and it is plausible that they vary significantly among countries. Nevertheless, the statistical strength of the association between the two metrics (asbestos consumption and mesothelioma mortality) has been repeatedly demonstrated [3].

Asbestos mineral type (and the fraction of specific mineral types in the total asbestos consumption) has been repeatedly demonstrated as one of the major predictors of mesothelioma risk [21,22]. In particular, the five amphibole minerals – actinolite, amosite, anthophyllite, crocidolite, and tremolite presented a carcinogenicity definitively higher than chrysotile and, for mesothelioma, the potency factor differences between amphibole and chrysotile is at least of three order of magnitude [23]. Our findings cannot consider this critical issue (according to the absence of adequate data) and this must be considered in their interpretation. In particular, it could be the reason for comparatively higher mesothelioma mortality in the UK, according to the extensive use of the amosite variety of asbestos [23]. In addition to the above limitation, the amount of asbestos consumption is affected by high uncertainty, as well as its yearly variation, in particular for developing countries. This poses problems in the assessment of a relationship with mesothelioma mortality, introducing high heterogeneity in the country specific exposure-lag-response function. Furthermore, an ecological study is probably an inappropriate tool for investigating the risk of mesothelioma by age. Analytical studies (in particular, cohort or cross sectional studies) seem certainly more appropriate to evaluate the trend in the relative risk by age and previous studies have demonstrated that mesothelioma risk depends non-linearly on age, based on occupational and residential cohort of exposed [24,25,26]. The United States Environmental Protection Agency (EPA) estimated that mortality rates from mesothelioma increases linearly with the intensity of exposure and, for a given intensity, increases with the time since first exposure [27]. Furthermore, the role of asbestos fibres dimensions in the carcinogenicity of amphibole must to be considered and the potency of fibres depends on mineralogical types and sizes [28,29,30], information that is not present in our study.

The lack of data from countries with the highest consumption, such as China, India, and Russia, represents another limitation in the assessment of the risks and impacts related to asbestos.

In China, asbestos has been largely used in the construction sector and asbestos-cement building material, but the presence of asbestos has been further documented in friction products, textiles, and insulation. Over the last twenty years, the Chinese Government has imposed some restrictions, such as the ban on amphibole asbestos imports and use, on production of asbestos-containing automotive friction materials, and on the use of all types of asbestos in siding and wall construction material [31]. In India, historically most of the asbestos was imported, but local sources from small-scale mining operations are also present. There is a lack of published studies about the modalities of industrial use of asbestos in India, but the mining industry and ship-breaking yards are probably sectors substantially involved in asbestos exposure for workers and citizens [31]. In line of principle, for the above three high consumption countries, this study could estimate the mesothelioma cases from the historical exposures by using the exposure, lag, and age functions provided by the meta-analysis. However, the high heterogeneities in the country specific exposure, lag, and age response functions did not suggest a direct transferability of the meta-analysis curves to other countries not included in the main analysis, and consequently their reliable estimation of mesothelioma deaths.

At the present, more than 60 countries have implemented a national ban on asbestos production and use. However, the only way to eliminate asbestos-related diseases globally is an international ban. Reliable estimations of asbestos use and its impact on public health could be important for countries without systematic production of data by national registries or mortality sources. The reasons for not banning asbestos are generally reported as scarcity of real and practicable alternatives, the risk of negative economic effects (particularly unemployment) and the strength of asbestos-producer lobbies in supporting the ‘safe use’ of asbestos theories. This study can contribute to counteracting such aspects by providing evidence of the huge impact of asbestos use on health and how this impact is distributed in time.

In conclusion, there is a need to improve the global epidemiological surveillance of asbestos-related diseases, particularly mesothelioma mortality, and the absence of reliable data for some major asbestos-user countries is a real concern. The analysis of historical asbestos consumption is a precious tool to verify the level of concordance with health effects figures in an international comparative approach. A reliable assessment of mesothelioma mortality is a fundamental step towards increasing the awareness of related risks and the need of an international ban on asbestos.

## Additional File

The additional file for this article can be found as follows:

10.5334/aogh.4012.s1 Supplemental Material. Figures SM1 and SM2.
