# Supplementary material for: Asbestos Consumption and Malignant Mesothelioma Mortality Trends in the Major User Countries
Source: Ann Glob Health. 2023 Feb 13;89(1):11. doi: 10.5334/aogh.4012 (PMC9936915; doi:10.5334/aogh.4012)

Supplemental material.

Figure SM1. Exposure–lag–response association between the relative risk (RR) of mesothelioma mortality and the past asbestos exposure. Pooled results from a meta-analysis of country-specific results.

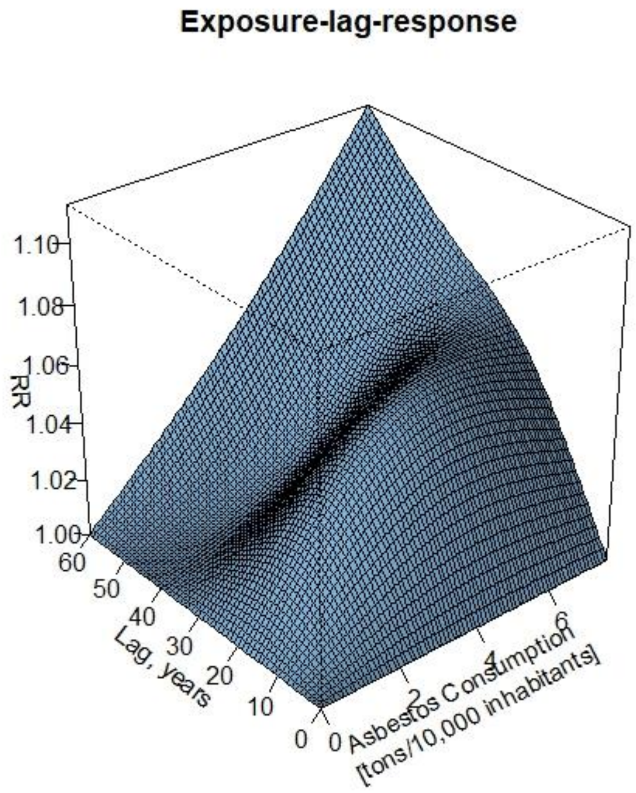

Figure SM2. Sensitivity analysis of the pooled meta-analysis of exposure, lag, and age response curves for different parameters of natural spline functions. Asbestos consumption at lag 30 years (a and c), lag at 3.9 tons/10,000 inhabitants of asbestos exposure (b and d) and age (e). Exposure and lag response curves for different degrees of freedom (d.f.) of the exposure function (a and b), fixing the d.f. of lag at 3. Exposure and lag response curves for d.f. of the lag function (c and d), fixing the d.f. of exposure at 2. Age response curve (e) for different knots of the age function (1 (age 65); 2 (40, 60); 3 (30, 50, 70)). The 95% confidence intervals are based on results of the main analysis (red curves).

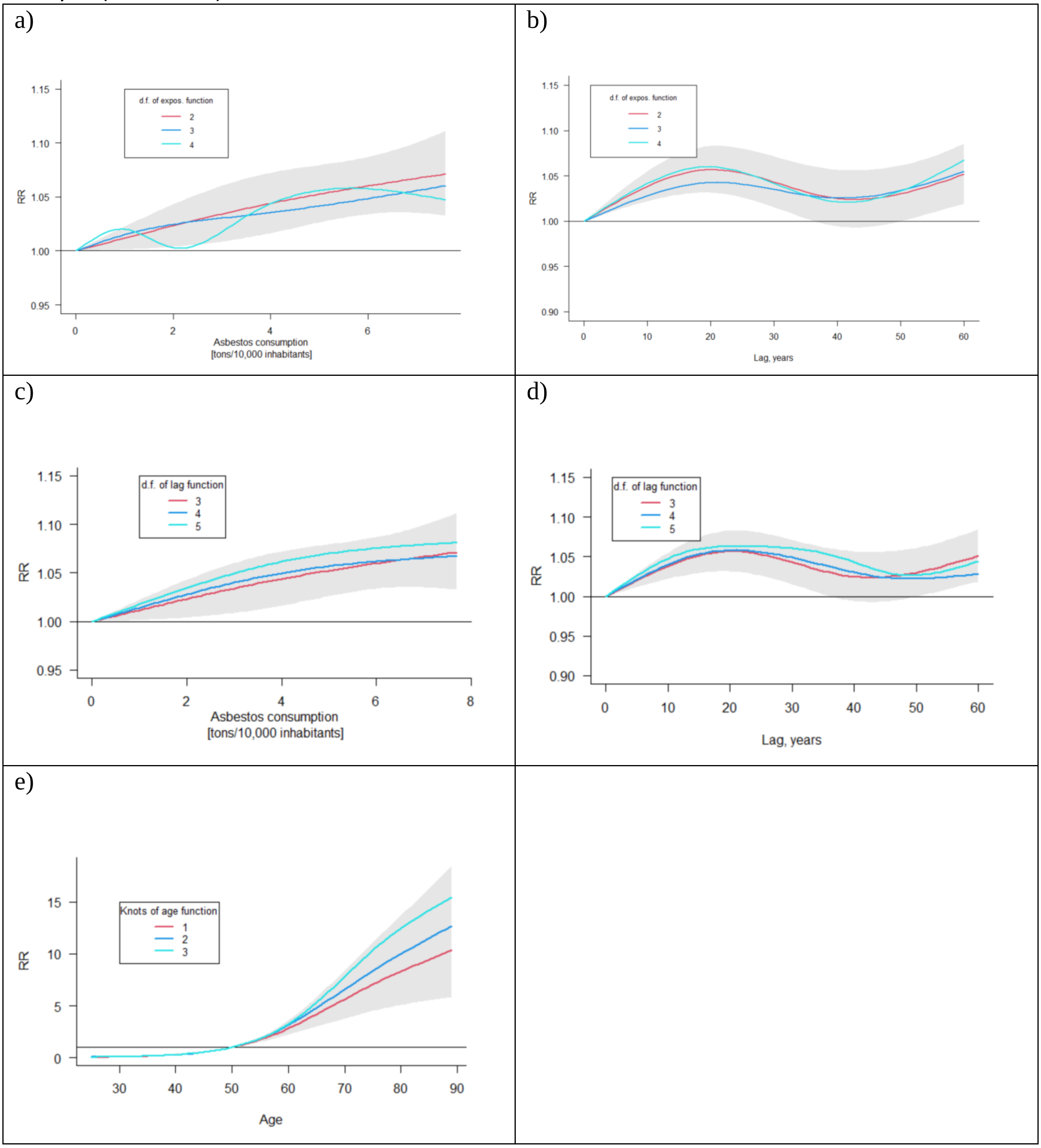

Supplement: Supplemental Material. — Figures SM1 and SM2. [file agh-89-1-4012-s1.pdf]
